# The anti-inflammatory Annexin A1 induces the clearance and degradation of the amyloid-β peptide

**DOI:** 10.1186/s12974-016-0692-6

**Published:** 2016-09-02

**Authors:** Miriam Ries, Rodrigo Loiola, Urvi N. Shah, Steve M. Gentleman, Egle Solito, Magdalena Sastre

**Affiliations:** 1 Division of Brain Sciences, Hammersmith Hospital, Imperial College London, London, W12 0NN UK; 2 William Harvey Research Institute, Barts and The London School of Medicine and Dentistry, Queen Mary University of London, Charterhouse Square, London, EC1M 6BQ UK

**Keywords:** Inflammation, Annexin A1, Alzheimer’s disease, Microglia, Amyloid-β, Anti-inflammatory, Neprilysin, Formyl-peptide receptor

## Abstract

**Background:**

The toxicity of amyloid-β (Aβ) peptide present in the brain of Alzheimer’s disease (AD) patients is thought to be mediated via the increased secretion of pro-inflammatory mediators, which can lead to neuronal dysfunction and cell death. In addition, we have previously shown that inflammation can affect Aβ generation. More recently, we have reported that in vitro administration of the anti-inflammatory mediator Annexin A1 (ANXA1) following an inflammatory challenge suppressed microglial activation and this effect was mediated through formyl peptide receptor-like 1 (FPRL1/FPR2) signalling. The aim of this study was to determine the potential role of ANXA1 in the generation and clearance of Aβ.

**Methods:**

We first compared ANXA1 protein expression in the brains of AD patients and healthy controls as well as in the 5XFAD model of AD. To determine the role of ANXA1 in the processing of amyloid precursor protein (APP) and the degradation of Aβ, N2a neuroblastoma cells were treated with human recombinant ANXA1 or transfected with ANXA1 siRNA. We also investigated the effect of ANXA1 on Aβ phagocytosis and microglial activation in BV2 cells treated with synthetic Aβ.

**Results:**

Our data show that ANXA1 is increased in the brains of AD patients and animal models of AD at early stages. ANXA1 was able to reduce the levels of Aβ by increasing its enzymatic degradation by neprilysin in N2a cells and to stimulate Aβ phagocytosis by microglia. These effects were mediated through FPRL1 receptors. In addition, ANXA1 inhibited the Aβ-stimulated secretion of inflammatory mediators by microglia.

**Conclusions:**

These data suggest that ANXA1 plays a pivotal role in Aβ clearance and supports the use of ANXA1 as potential pharmacological tool for AD therapeutics.

**Electronic supplementary material:**

The online version of this article (doi:10.1186/s12974-016-0692-6) contains supplementary material, which is available to authorized users.

## Background

Amyloid-β (Aβ) peptide is present in high levels in the brains of Alzheimer’s disease (AD) patients and is closely associated with the pathogenesis of the disease. Aβ peptides are toxic products derived from the catalytic cleavage of a larger amyloid precursor protein (APP) by β- and γ-secretases [1]. The toxicity of Aβ is thought to be mediated via the secretion of neurotoxic inflammatory mediators and reactive oxygen species (ROS) from glial cells [2]. There is convincing evidence that Aβ is able to prime microglia [3], inciting an inflammatory response and the release of neurotoxic cytokines, ROS, complement factors and nitric oxide (NO), which can all contribute to neuronal dysfunction and cell death [4]. Importantly, we previously found a direct link between pro-inflammatory cytokines and Aβ generation by showing that certain cytokines such as tumour necrosis factor (TNF)α and interferon (IFN)γ can transcriptionally upregulate β-secretase beta-site APP cleaving enzyme 1 (BACE1) [5, 6]; this has also been confirmed in animal models of inflammation [7]. Therefore, a combination of dying neurones and Aβ accumulated via active synthesis would further stimulate microglia, creating a self-perpetuating cycle. It has to be noted that the activation of microglia may not only contribute to disease progression but could also have beneficial effects. Activated microglia can reduce Aβ accumulation by increasing its phagocytosis, clearance and degradation [8, 9] and the release of anti-inflammatory molecules including certain cytokines, growth factors and the resolving molecule Annexin A1 (ANXA1).

ANXA1 is a glucocorticoid anti-inflammatory mediator in the peripheral system [10, 11], which plays a key role in ensuring the effective and selective removal of apoptotic neuron-like cells under inflammatory and non-inflammatory conditions [12]. In the brain, ANXA1 is abundant in microglial cells and in the endothelium of the blood brain barrier (BBB), where it plays an important role in maintaining BBB tightness [13, 14]. Microglia have the capacity to synthesise and release ANXA1 [15], and ANXA1 function is associated with anti-inflammatory actions, regulating leukocyte extravasation [16–18], macrophage phagocytosis [19] and glucocorticoid action [20–22]. During pathological states, it has been proposed that ANXA1 has a protective role by limiting inflammatory damage [23]. This was further supported by our observations whereby incubation of microglia with recombinant ANXA1 resulted in reduced microglial activation following lipopolysaccharide (LPS) stimulation [12]. Our studies have also shown that ANXA1 is upregulated in human microglia surrounding Aβ plaques, supporting a possible role for the protein in regulating the microglial response to amyloid plaques [12, 15]. The identification of formyl peptide receptor-like 1 (FPRL1/FPR2) as a receptor for ANXA1 suggests an intriguing link between Aβ and FPRL1 [24]. The binding of ANXA1 to FPRL1 has been associated with the modulation of microglial phagocytosis [12, 25] and pro-inflammatory release [26], while it was suggested that the interaction of different aggregated/fibrillar forms of Aβ with microglia changes the expression pattern of FPRL1, affecting the phagocytic function of microglia [27].

We therefore hypothesize﻿d that ANXA1 has a protective role in resolving neuroinflammation in the AD brain, by affecting Aβ generation and/or degradation and modulating microglial functions such as phagocytosis and the secretion of cytokines and neurotoxic species, which could cause neuronal damage. The aim of this study was to define the role of ANXA1 in APP processing and Aβ degradation in vitro and to evaluate whether ANXA1 is able to affect Aβ-induced changes in microglia function.

## Methods

### Reagents and antibodies

Antibodies used included 6E10 against Aβ1-16 (Covance), 4G8 against Aβ17-24 (Covance), anti-BACE1 antibody (Cell Signalling), anti-neprilysin antibody (Santa Cruz), anti-insulin degrading enzyme (IDE) antibody (Abcam), anti-ANXA1 antibody (Zymed), anti-β-actin antibody (Abcam), anti-IgG antibody (Abcam), anti-FPRL1/FPR2 antibody (Acris Antibodies GmbH) and anti-IgG FITC conjugated antibody (AbD Serotec). Full-length human recombinant (hr) ANXA1 was obtained as previously described [16], and protein was purified by GTP Technology (Labege Cedex). Synthetic Aβ_1–42_, 5-FAM-labelled Aβ_1–42_ and Aβ_1–42_ scrambled peptides were obtained from Anaspec. Synthetic Aβ_1–42_ was prepared by suspension of the lyophilised Aβ_1–42_ in DMSO to 500 μM and then diluted to different concentrations ranging from 0.1 to 3 μM with cold DMEM. FPR2 inhibitors WRW4 and Boc-1 were obtained from Tocris Bioscience. Tissue culture reagents were obtained from Invitrogen. All other chemicals and reagents were purchased from Sigma-Aldrich, Qiagen and Invitrogen.

### Human postmortem brain samples

Human brains were obtained from routine autopsies at the London Neurodegenerative Diseases Brain Bank at King’s College London and the Swedish Brain Bank in accordance with laws and the permission of the ethical committees. The control group included brains from subjects who died either of non-neurological diseases or traffic accidents and had no history of long-term illness or dementia. Frontal cortex was compared of control brains (*n* = 20, 12 males, 8 females, range 40–97 years, mean age 73.24 ± 4 years) and sporadic AD cases (*n* = 22, 11 males, 11 females, range 42–98 years, mean age 79.09 ± 3 years).

### Immunohistochemistry

For immunohistochemistry, sections were deparaffinised in xylene and rehydrated in decreasing alcohols. Endogenous peroxidase was inhibited by incubation in 0.3 % *v*/*v* H_2_O_2_ in 0.1 M phosphate buffered saline (PBS) for 30 min at room temperature. Sections were rinsed twice in 0.05 M Tris-buffered saline (TBS) and incubated for 1 h at room temperature with 10 % BSA (Sigma-Aldrich) containing 0.05 % *v*/*v* Triton X-100 to saturate nonspecific binding; sections were then incubated for 16 h at 4 °C with ANXA1 antibody (1:1000), rinsed twice in TBS-containing 0.05 % *v*/*v* Triton X-100, incubated in biotinylated goat-anti-rabbit secondary antibody (Vector Laboratories) at the dilution of 1:100 for 2 h at room temperature, rinsed twice with TBS and incubated for 45 min in the avidin-biotin complex (ABC) conjugated with horseradish peroxidase (HRP) in TBS (Vector Laboratories). Following two washes in TBS, the reactions were developed in 0.025 % diaminobenzidine and 0.01 % H_2_O_2_ (Sigma-Aldrich) in TBS for 5 min. Sections were rinsed twice in TBS, dehydrated and mounted under DPX mountant (VWR International) for bright-field microscopic analysis.

### Animals

All animals were kept in individually ventilated cages with controlled temperature and humidity, food and water ad libitum and a 12:12-h light-dark cycle. Twelve- and 26-month-old male 5XFAD transgenic mice and their wild-type littermates (*n* = 6 per group) were used and were obtained from the Jackson Laboratory. These mice overexpress both mutant human APP(695) with the Swedish (K670N, M671L), Florida (I716V) and London (V717I) Familial Alzheimer’s Disease (FAD) mutations and human PS1 harbouring two FAD mutations, M146L and L286V. Expression of both transgenes is regulated by neural-specific elements of the mouse Thy1 promoter to drive overexpression in the brain. 5XFAD mice generate almost exclusively Aβ_42_ and rapidly accumulate massive cerebral levels [28]. Animals were anaesthetised and transcardially perfused with ice-cold 0.9 % saline. Brains were dissected and snap frozen and were stored at −80 °C until used. All animal procedures were approved by the UK Home Office and were in accordance with the Animals (Scientific Procedures) Act of 1986.

### Cell lines and maintenance

The murine neuroblastoma cell line stably transfected with the APP “Swedish” mutation (K595N/M596L), hereafter referred to as N2asw, was a kind gift of G. Thinakaran (University of Chicago). Cells were maintained in a selective and undifferentiated state using 0.2 mg/ml of the antibiotic G-418 in DMEM and Opti-MEM (Gibco/Life Technologies), supplemented with 5 % foetal bovine serum (FBS) and 50 U/ml penicillin, and 50 mg/ml streptomycin sulphate. The human neuroblastoma cell line SK-N-SH was cultured in DMEM supplemented with 10 % FBS, 100 U/ml penicillin and 100 mg/ml streptomycin sulphate. The murine microglial cell line BV2 was cultured in Roswell Park Memorial Institute (RPMI) medium containing 5 % FBS, gentamycin (50 mg/ml) and 1 % non-essential amino acids (NEAA). Cells were grown in a 5 % CO_2_ incubator at 37 °C.

### Treatments, transfection and infection

Cells were incubated with different concentrations of human recombinant ANXA1 (hrANXA1), ranging from 0.04 to 5 μg/ml for 2–48 h in serum-free medium. In some experiments, hrANXA1 treatment was combined with 0.1–3 μM synthetic Aβ_42_ (Anaspec), non-selective FPR inhibitor Boc1 (5 μg/ml) or FPR2 inhibitor WRW4 (0.5–5 μM), with hrANXA1 added 30 min before Aβ and Boc1 or WRW4 added 20 min before hrANXA1. For fluorescence-activated cell sorting (FACS) experiments, BV2 cells were left in medium (containing FBS) which they had conditioned overnight for 18 h, or this was changed to fresh medium (containing FBS), prior to addition of 5-FAM-labelled-Aβ_42_ or Aβ_42_ scrambled (3 μg/ml) and anti-ANXA1-antibody (20 ng/ml) or anti-IgG-antibody (20 ng/ml) for 3 h. For Image Stream experiments, cells were incubated with 5-FAM-labelled-Aβ_42_ or Aβ_42_ scrambled (5 μg/ml), with 5 μg/ml hrANXA1 added 30 min before Aβ and 5 μM WRW4 added 20 min before hrANXA1.

N2asw cells were transfected with control or ANXA1 small interfering RNA (siRNA) (Gene Pharma) using Lipofectamine® 2000 transfection reagent (Thermo Fischer Scientific) according to the manufacturer’s instructions and harvested after 48 h.

BV2 cells were plated in six-well plates (300,000 cells/well). After 48 h, when having reached 70–80 % of confluence, cells were incubated for 18 h overnight at 37 °C with medium containing lentivirus small hairpin RNA (shRNA) murine ANXA1 with designated specific clones named TRCN0000109725(A) and TRCN0000109728 (B) as previously described [29]. Mock-infected cells (PKCO) were used as control of infection procedure. Transduction efficiency and ANXA1 knockdown were verified by Western blot analysis (Additional file 1: Figure S1).

### Analysis of Aβ and sAPPα

Soluble APPα (sAPPα) and Aβ secreted in the conditioned medium of N2asw cells were analysed by Western blot. The volume of medium used was adjusted to protein concentrations measured in total cell lysates. An aliquot of the media was either run straight away on NuPage 4–12 % Tris-glycine gel (Invitrogen) or Aβ was pulled down overnight at 4 °C using Sepharose Protein A (Zymed) and 4G8 (Covance). Samples were then loaded in NuPage 4–12 % Tris-glycine gels and transferred onto nitrocellulose membranes. The membrane was boiled in PBS for 5 min, blocked with non-fat milk and incubated with 6E10 antibody at 1/1000 (a monoclonal antibody recognising amino acids 1–17 of human Aβ (Covance)). Membranes were incubated with HRP conjugated secondary in 5 % non-fat dried milk in Tris-buffered saline with Tween (TBST) and developed using ECL™ (GE Amersham) and Hyperfilm ECL™ (GE Amersham) in an automated developer from Konica, SRX 101A.

### Western blotting analysis

Cell lysates and brain homogenates were extracted with RIPA (1 % Triton X-100, 1 % sodium deoxycholate, 0.1 % SDS, 150 mM NaCl, and 50 mM Tris-HCl, pH 7.2) supplemented with cOmplete™ protease inhibitor cocktail (Roche), and equal amounts of protein samples (20–80 μg) were separated in SDS-PAGE gels, followed by immunoblotting with primary antibodies and detected with HRP conjugated secondary in 5 % non-fat dried milk or 5 % bovine serum albumin (BSA) in TBST. Membranes were developed using ECL™ (GE Amersham) reagents and using Hyperfilm ECL™ in an automated developer from Konica, SRX 101A. To re-probe blots for a different protein, membranes were stripped with ReBlot Plus Strong Antibody Stripping Solution (Millipore). Digital images were quantified by densitometry using ImageJ and adjusted for protein loading by normalising to β-actin, GAPDH or tubulin, or full-length APP for APP cleavage products.

### β-secretase activity assay

β-secretase enzyme activity was measured in N2asw cell lysates using a fluorimetric reaction (Abcam) according to the manufacturer’s instructions.

### Neprilysin activity assay

Treated cells were collected and resuspended in 10 mM Tris-HCl pH 7.5 and incubated with 100 mM substrate *N*-succinyl alanin-alanin-phenylalanin-7-amino-4-methylcoumarin (Sigma) for 30 min at 37 °C. Fluorescence was measured at 390-nm excitation and 420-nm emission wavelength, and results were adjusted to protein concentration [30].

### qPCR

Messenger RNA (mRNA) was extracted from treated cells using the Direct-zol RNA Mini Prep system (Zymo Research) according to the manufacturer’s instructions. mRNAs were subjected to reverse-transcription quantitative PCR (RT-qPCR) analysis using a two-step method with an initial RT and subsequent real-time cycling as reported previously [30] on a Stratagene Mx3000p block cycler. *GAPDH*/*Gapdh* was used as an internal control for mRNA. Primers used are listed in Table 1.

**Table 1:** List of primers used

Gene	Forward	Reverse
*Arg1*	CAGCACTGAGGAAAGCTGGT	CAGACCGTGGGTTCTTCACA
*BACE1*	GGCGGGAGTGGTATTATGAGGTGA	TATTGCTGCGGAAGGATGGTGA
*FPR rs1*	CCTTGGACCGCTGTATTTGT	GTGCACATCCCCTCTAGCAT
*Gapdh*	ACCACAGTCCATGCCATCAC	TCCACCACCCTGTTGCTGTA
*GAPDH*	AGGGCTGCTTTTAACTCTGGT	CCCCACTTGATTTTGGAGGGA
*Il10*	TAACTGCACCCACTTCCCAG	AGGCTTGGCAACCCAAGTAA
*Il4*	GGTCTCAACCCCCAGCTAGT	GCCGATGATCTCTCTCAAGTGAT
*Il6*	ATGGATGCTACCAAACTGGAT	TGAAGGACTCTGGCTTTGTCT
*MARCO*	ACAGAGCCGATTTTGACCAAG	CAGCAGTGCAGTACCTGCC
*MME*	GGTCCGCAGCTAAGGTCCAG	GAGCTGGTCTCGGGAATGAC
*RAGE*	CTTGCTCTATGGGGAGCTGTA	GGAGGATTTGAGCCACGCT
*Tgfb1*	GGATACCAACTATTGCTTCAGCTCC	AGGCTCCAAATATAGGGGCAGGGTC
*Tnf*	AGGGATGAGAAGTTCCCAAATG	CACTTGGTGGTTTGCTACGAC

### Fluorescence-activated cell sorting

BV2 microglial cells were plated in a 12-well plate overnight in RPMI containing 5 % FBS. The following day, the medium was either replaced with RPMI containing 1 % FBS or unchanged, and the cells were then incubated with 3 μg/ml of 5-FAM-labelled Aβ_1–42_ or 5-FAM-labelled scrambled-Aβ_1–42_, for 3 h. Following this, the medium was removed and the cells were washed with cold PBS and fixed with 2 % paraformaldehyde (PFA) for 10 min. After this, cells were washed and collected for analysis by FACS using FACSCalibur (Becton, Dickinson and Company) with a 100-mW, 488-nm, air-cooled argon laser. Data was measured from the FL1 channel (mean intensity of fluorescence in log scale) with at least 10,000 events counted and analysed using FlowJo software.

### Image stream

BV2 cells (wild type, shRNA PKCO and shRNA ANXA1 492) were plated in sixwell plates. After 24 h, cells were incubated for 3 h at 37 °C with 5 μg/ml of 5-FAM-labelled Aβ_1–42_ added to BV2-conditioned medium. In order to investigate the effect of ANXA1 on Aβ_1–42_ phagocytosis, cells were pre-incubated with hrANXA1 (5 μg/ml) 30 min prior incubation with 5-FAM-labelled Aβ_1–42_. To further evaluate the involvement of FPR2 on Aβ_1–42_ phagocytosis, cells were pre-incubated with WRW4 (5 μM) 20 min prior to incubation with hrANXA1. Following phagocytosis, cells were washed three times with cold PBS and detached from plates by using trypsin (0.20 %). Cells collected were fixed with 2 % PFA for 10 min at room temperature. Imaging flow cytometry was performed on an ImageStreamx Mark II operated by INSPIRE software (Amnis Corporation). The 5-FAM-labelled Aβ_1–42_ fluorescence was recorded using excitation with a 488-nm laser at 50-mW intensity and emission collected with a 480–560-nm filter in the camera 2 (CH2), while bright-field images were collected in the cameras 1 (CH1) and 9 (CH9). A sample of BV2 cells that were not incubated with 5-FAM-labelled Aβ_1–42_ (control—negative phagocytosis) was collected at the same settings, in order to gate different cell populations (negative or positive phagocytosis). Image-based gating was performed following the method reported in detail in Additional file 2 and Additional file 3: Figure S3. In each experiment, a template of settings used to analyse control cells was created and was applied to all files. A total of 10,000 events were collected for each sample, and data were analysed using IDEAS Application 6.1 software (Amnis Corporation).

### Statistical analysis

Data shown are mean ± standard error of the mean (SEM). Statistical analysis was performed using GraphPad Prism 5 software. One- or two-way analysis of variance (ANOVA) with Bonferroni’s multiple-comparison post hoc test were used, or when appropriate, a Kruskal-Wallis test with Dunn’s multiple-comparison post-test or an independent two-tailed Student’s *t* test, with a value of *p* < 0.05 being considered statistically significant.

## Results

### ANXA1 is increased in postmortem brains of AD patients and in 5XFAD mice

**Fig. 1:**
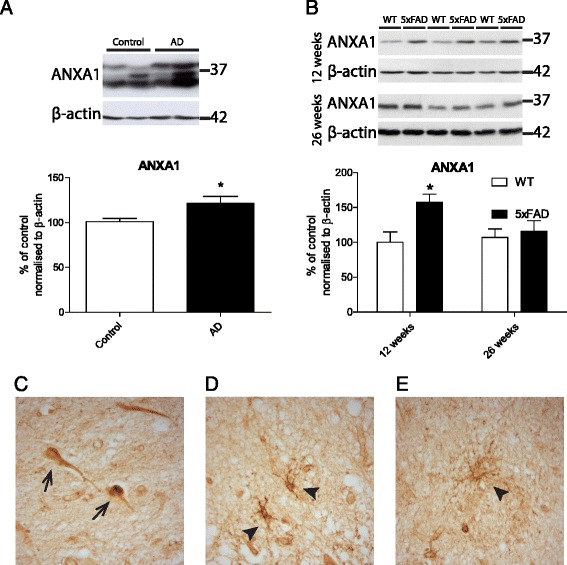
ANXA1 is increased in postmortem brains of AD patients and in 5XFAD mice. **a** Representative blots and quantification of ANXA1 expression in the frontal cortex of neurologically healthy controls and sporadic Alzheimer’s patients and normalised to β-actin (*n* = 20 controls, 12 males, 8 females, range 40–97 years, mean age 73.24 ± 4 years, *n* = 22 AD cases, 11 males, 11 females, range 42–98 years, mean age 79.09 ± 3 years). **b** Representative blots and quantification of ANXA1 expression in the cortex of 5XFAD mice and wild-type littermates and normalised to β-actin (*n* = 6/group, males aged 12 and 26 weeks). **c**–**e** Representative images of human postmortem AD brain hippocampal sections stained for ANXA1. *Arrows* indicate expression in neurons (**c**), microglia (**d**) and astrocytic (**e**) staining. Values shown in graphs represent the mean value ± SEM and are expressed as fold change in comparison to the normalized control. Statistical analysis included Student’s independent two-tailed *t* test, *p < 0.05

ANXA1 expression was determined in homogenates of human postmortem frontal cortex from AD patients and in age-matched healthy control brains. The results obtained from Western blotting analysis revealed an increase of ~20 % in the expression of ANXA1 (37 kDa) in AD brains compared with matched controls (*n* = 21 controls and 22 sporadic AD cases, Fig. 1a). Supporting data obtained in human AD cases, a 50 % increase in ANXA1 expression was also observed in brain homogenates of 5XFAD mice at 12 weeks of age (*n* = 6/group, Fig. 1b). However, at 26 weeks of age, when the animals show extensive amyloid plaque deposition, there were no significant differences in ANXA1 levels between 5XFAD mice and wild-type controls. We did not observe changes in FPRL1/FPR2 expression in either AD patients (Additional file 4: Figure S4A) or animal models (Additional file 4: Figure S4B).

In addition, immunostaining of ANXA1 in hippocampal sections from AD patients showed neuronal (Fig. 1c) as well as microglial (Fig. 1d) and astrocytic (Fig. 1e) staining. Intriguingly, ANXA1 was highly expressed in neuronal tangles (marked by arrows), indicating a potential role of ANXA1 in neuronal pathology in AD.

### Treatment of neuroblastoma cells with recombinant ANXA1 reduces the levels of soluble Aβ

**Fig. 2:**
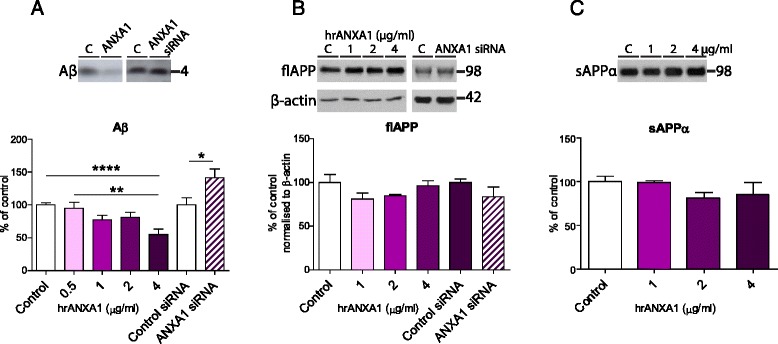
Treatment of neuroblastoma cells with recombinant ANXA1 reduces the levels of soluble Aβ. **a** Representative Western blots and quantification of soluble Aβ in the medium of N2asw cells treated with 0.5–4 μg/ml hrANXA1 for 18 h (*n* = 9–24 samples) or transfected with control or ANXA1 siRNA (*n* = 15 samples). **b** Representative Western blots and quantification of full-length APP protein expression in N2asw cells treated with 1–4 μg/ml hrANXA1 for 18 h (*n* = 3–11 samples) or transfected with control or ANXA1 siRNA (*n* = 15 samples) and normalised to β-actin. **c** Representative Western blots and quantification of soluble APPα in the medium of N2asw cells treated with 1–4 μg/ml hrANXA1 for 18 h (*n* = 3–11 samples). Values shown in graphs represent the mean value ± SEM and are expressed as fold change in comparison to the normalized control. Statistical analysis included one-way ANOVA with Bonferroni’s multiple-comparison post-test or independent two-tailed Student’s *t* test, *p < 0.05, **p < 0.01, ****p < 0.0001

Because ANXA1 is expressed by neurons (Fig. 1c), we investigated the effect of ANXA1 on APP processing in a neuroblastoma cell line. Consequently, N2asw cells were incubated for 18 h with different concentrations of hrANXA1 ranging from 0.5 to 4 μg/ml (corresponding to 13 to 105 nM). Our data show that hrANXA1 induced a concentration-dependent decrease in the levels of Aβ in the media of these cells (Fig. 2a). Conversely, ANXA1 knockdown using siRNA transfection resulted in an increase in Aβ secretion (Fig. 2a).

**Fig. 3:**
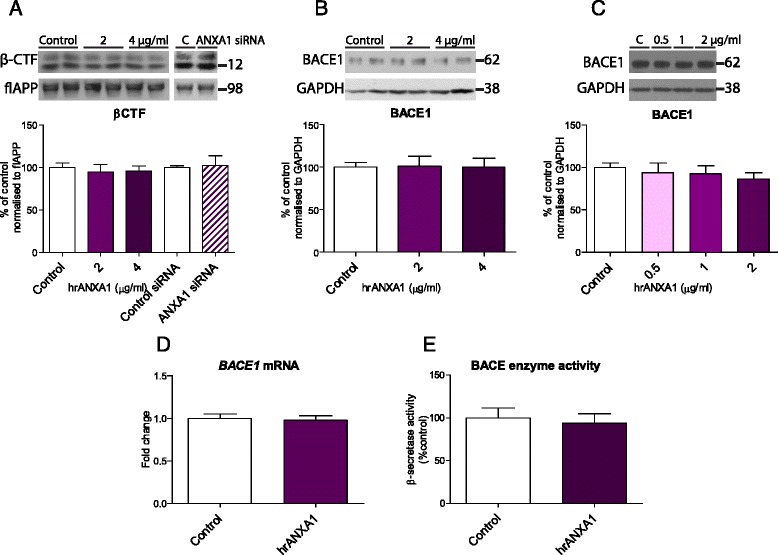
Treatment of neuroblastoma cells with recombinant ANXA1 does not affect BACE1 activity or expression. **a** Representative Western blots and quantification of β-CTF expression in N2asw cells treated with 2–4 μg/ml hrANXA1 for 18 h (*n* = 9–18 samples) or transfected with control or ANXA1 siRNA (*n* = 9 samples) and normalised to full-length APP. **b** Representative Western blots and quantification of BACE1 protein expression in N2asw cells treated with 2–4 μg/ml hrANXA1 for 18 h and normalised to GAPDH (*n* = 6–12 samples). **c** Representative Western blots and quantification of BACE1 protein expression in SK-N-SH cells treated with 0.5–2 μg/ml hrANXA1 for 18 h and normalised to GAPDH (*n* = 15–27 samples). **d** Quantification of *BACE1* mRNA expression by qPCR analysis in SK-N-SH cells treated with 2 μg/ml hrANXA1 for 18 h (*n* = 9 samples). **e** Quantification of β-secretase activity in N2asw cells following treatment with 4 μg/ml hrANXA1 for 18 h (*n* = 5 samples). Values shown in graphs represent the mean value ± SEM and are expressed as fold change in comparison to the normalized control

The reduction in Aβ was not secondary to alterations in the expression of APP or the processing of APP by α-secretase, as no changes were detected in the expression of full-length APP in cell lysates (Fig. 2b) or in sAPPα in the conditioned medium (Fig. 2c). To determine whether incubation with recombinant ANXA1 decreased Aβ by altering the amyloidogenic pathway, the levels of β-carboxy-terminal-fragments (CTFs), as measurement of β-secretase activity, were quantified, showing no changes with hrANXA1 treatment (Fig. 3a). Similar results were obtained performing an in vitro β-secretase assay (Fig. 3e). Additionally, no effects on the BACE1 protein and *BACE1* mRNA expression by treatment with hrANXA1 (0.5–2 μg/ml) were confirmed in N2asw and in human neuroblastoma SK-N-SH cells (Fig. 3b–d).

Therefore, these results suggest that ANXA1 reduces Aβ levels in neurons, and this effect is not mediated by changes in the expression or enzymatic cleavage of APP.

### hrANXA1 increases the expression and activity of Aβ-degrading enzyme neprilysin

**Fig. 4:**
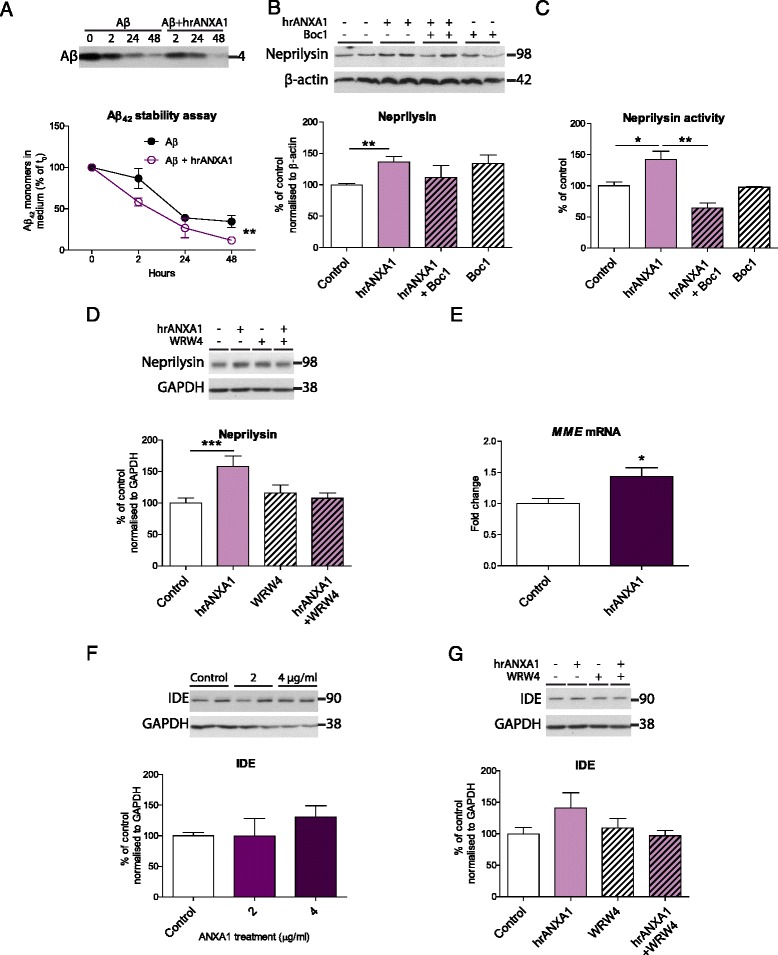
hrANXA1 treatment increases the expression and activity of Aβ-degrading enzyme neprilysin. **a** Representative Western blots and quantification of synthetic Aβ_1–42_ monomers expression in the medium of N2asw cells incubated with synthetic Aβ_1–42_ (0.1 μM), following 0–48-h treatment with 10-nM hrANXA1 assessed by Western blotting using 6E10 antibody (*n* = 3). **b** Representative Western blots and quantification of neprilysin protein expression in N2asw cells treated with 4 μg/ml hrANXA1 and/or the non-selective FPR inhibitor Boc1 (5 μg/ml) for 18 h, normalised to β-actin (*n* = 10–37 samples). **c** Quantification of neprilysin activity in N2asw cells treated with 4 μg/ml hrANXA1 and/or the non-selective FPR inhibitor Boc1 (5 μg/ml) for 18 h (*n* = 2–8 samples). **d** Representative Western blots and quantification of neprilysin protein expression in SK-N-SK cells treated with 2 μg/ml hrANXA1 and/or selective FPR2 inhibitor WRW4 (5 μM) for 18 h normalised to GAPDH (*n* = 11–21 samples). **e** Quantification of *MME* (neprilysin) mRNA expression by qPCR analysis in SK-N-SK cells treated with 2 μg/ml hrANXA1 for 18 h (*n* = 5–6 samples). **f** Representative Western blots and quantification of IDE protein expression in N2asw cells treated with 2–4 μg/ml hrANXA1 for 18 h and normalised to GAPDH. **g** Representative Western blots and quantification of IDE protein expression in SK-N-SK cells treated with 2 μg/ml hrANXA1 and/or selective FPR2 inhibitor WRW4 (5 μM) for 18 h, normalised to GAPDH (*n* = 10–18 samples). Values shown in graphs represent the mean value ± SEM and are expressed as a percentage change in comparison to the normalized control or fold change of control. Statistical analysis included two-way ANOVA, one-way ANOVA with Bonferroni’s multiple-comparison post-test, Kruskal-Wallis test with Dunn’s multiple-comparison post-test or unpaired Student’s two-tailed *t* test as appropriate, *p < 0.05, **p < 0.01, ***p < 0.001

To understand the mechanism behind ANXA1’s effect on reducing Aβ levels in N2a cells we investigated the effect of ANXA1 on Aβ clearance. For this purpose, synthetic Aβ_1–42_ peptide (0.1 μM) was added to the medium of N2asw cells over 48 h with or without 10 nM hrANXA1 (Fig. 4a). In cells treated with hrANXA1, the levels of Aβ_1–42_ synthetic peptide were reduced at faster rate compared with control cells (two-way ANOVA, effect of treatment *F*(1,14) = 11.60, *p* = 0.0043; effect of time *F*(3,14) = 64.71, *p* < 0.0001) (Fig. 4a). These results show that the stability of synthetic Aβ peptide in the medium is affected in cells treated with hrANXA1.

We then examined whether stimulation with hrANXA1 induced an increase in enzymatic mechanisms of Aβ degradation. The expression levels of Aβ-degrading enzymes, insulin-degrading enzyme (IDE) and neprilysin were assessed by Western blot. Interestingly, 2-μg/ml hrANXA1 treatment of N2asw cells led to a significant increase in neprilysin expression (Fig. 4b), which paralleled with higher neprilysin activity levels (Fig. 4c). This effect was partially reversed by the non-selective FPR inhibitor Boc1 (5 μg/ml) (Fig. 4c), indicating that the effect might be mediated by the binding of ANXA1 to FPR receptors. Similar results were obtained in human SK-N-SH cells treated with 2-μg/ml hrANXA1, showing increased neprilysin expression, which was attenuated by the selective FPR2 inhibitor WRW4 (5 μM) (Fig. 4d). We had previously proven the expression of FPR in both types of cells (Additional file 4: Figure S4C-D). This effect was associated with an increase in neprilysin transcription, with quantitative RT-PCR experiments showing higher neprilysin gene (*MME*) expression in SK-N-SH cells treated with 2 μg/ml hrANXA1 (Fig. 4e). The levels of IDE however were non-significantly altered by 2–4 μg/ml hrANXA1 treatment in both mouse N2asw (Fig. 4f) and human SK-N-SH cells (Fig. 4g).

### hrANXA1 increases microglial phagocytosis of Aβ_1–42_

**Fig. 5:**
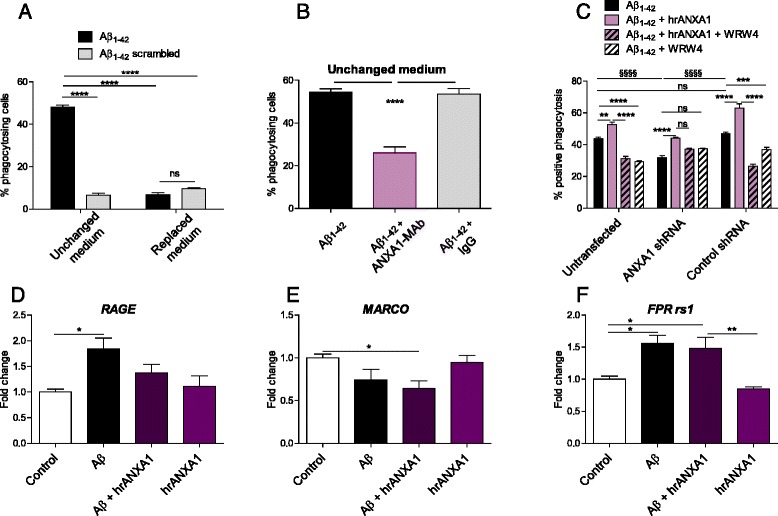
ANXA1 increases microglial phagocytosis of Aβ_1–42_. **a**–**b** Phagocytosis of 5-FAM-labelled-Aβ_1–42_ or 5-FAM-labelled-scrambled Aβ_1–42_ by BV2 microglia incubated for 3 h with 3 μg/ml of these compounds in medium that has been conditioned by BV2 cells for 18 h overnight or freshly changed prior to Aβ incubation (*n* = 9), measured by FACS. **b** Phagocytosis of 5-FAM-labelled-Aβ_1–42_ (3 μg/ml) by BV2 microglia after 3-h incubation with anti-ANXA1-antibody (20 ng/ml) or anti-IgG-antibody when medium has been conditioned by BV2 cells overnight, measured by FACS (*n* = 9). **c** Phagocytosis of 5-FAM-labelled-Aβ_1–42_ (5 μg/ml) incubated for 3 h with BV2 microglia (untransfected, control shRNA or ANXA1 shRNA transfected) in the absence or presence of hrANXA1 (5 μg/ml) and/or selective FPR2 antagonist WRW4 (5 μM) (*n* = 3–9). **d**–**f** Quantification of mRNA levels of receptors involved in receptor-mediated endocytosis in BV2 cells treated with 3 μM Aβ_1–42_ with or without 5 μg/ml hrANXA1 for 16 h. **d** *RAGE* (*n* = 6 samples). **e** *MARCO* (*n* = 6). **f** *FPR rs1* (*n* = 6). Values shown in graphs represent the mean value ± SEM and are expressed as a percentage change in comparison to the normalized control or fold change of control. Statistical analysis included Student’s independent two-tailed *t* test, one-way ANOVA with Bonferroni’s multiple-comparison post-test or two-way ANOVA with Bonferroni’s multiple-comparison post-test as appropriate. *p < 0.05, **p < 0.01, ***p < 0.001, ****/^§§§§^ *p* < 0.0001

Since microglia plays a crucial role in removing Aβ_1–42_ and we have previously shown that ANXA1 has an important role in efferocytosis or phagocytosis/phagoapoptosis [12], we tested the effects of endogenous ANXA1 on microglial phagocytosis of Aβ_1–42_ using fluorescently labelled Aβ_1–42_ or scrambled Aβ_1–42_ by FACS analysis. BV2 cells incubated with 5-FAM-labelled Aβ_1–42_ (3 μg/ml) for 3 h in medium that had been conditioned by BV2 cells overnight (unchanged media) showed higher phagocytic activity compared to 5-FAM-labelled Aβ_1–42_ (3 μg/ml, 3 h) added into fresh new media (Fig. 5a). This suggests that an endogenous molecule released by the microglia in unchanged media had a positive effect on enhancing Aβ_1–42_ phagocytosis. The specificity of this observation was shown by the lack of effect of ANXA1 on the phagocytosis of scrambled Aβ_1–42._ Since BV2 cells secrete ANXA1 into the supernatant [12], we next tested whether the factor released by microglia contributing to Aβ_1–42_ phagocytosis was indeed ANXA1. For this reason, 5-FAM-labelled Aβ_1–42_ phagocytosis was determined in BV2 cells with conditioned media including a neutralizing ANXA1 antibody (20 ng/ml) or IgG control antibody. The results in Fig. 5b show that treatment with anti-ANXA1-antibody-resulted in a reduction in 5-FAM-labelled Aβ_1–42_ phagocytosis, which was not detected with anti-IgG antibody control.

To further confirm the specificity of the effect of endogenous ANXA1 on Aβ_1–42_ phagocytosis, we tested the effect of ANXA1 knockdown on microglial phagocytosis of 5-FAM-labelled Aβ_1–42_. BV2 cells infected with shRNA lentivirus (wild type, PKCO, and shRNA ANXA1—clone 492) were incubated (3 h) with 5-FAM-labelled Aβ_1–42_ (5 μg/ml), and phagocytosis was measured using an imaging flow cytometer (ImageStream). The efficiency of the shRNA infection is shown in Additional file 1: Figure S1. As illustrated in Fig. 5c, the phagocytosis of 5-FAM-labelled Aβ_1–42_ was significantly reduced in ANXA1 knockdown cells in comparison to control BV2 cells and mock-infected BV2 cells. The representative histograms of image-based cytometry analysis of 5-FAM-labelled Aβ_1–42_ phagocytosis are shown in Additional file 3: Figure S3D.

Furthermore, we investigated the pharmacological effect of ANXA1 on Aβ_1–42_ phagocytosis, and BV2 cells were pre-incubated with 5 μg/ml of hrANXA1 30 min prior to incubation with 5-FAM-labelled Aβ_1–42._ Results presented in Fig. 5c show clearly the ability of the recombinant molecule to stimulate the phagocytosis of 5-FAM-labelled Aβ_1–42_, further supporting our hypothesis that ANXA1 stimulates the uptake of Aβ_1–42_ in a non-phlogistic manner.

Next, we explored the involvement of FPR2 on the ANXA1-mediated increase on Aβ_1–42_ phagocytosis. BV2 cells were pre-incubated with WRW4, a specific antagonist of FPR2, 20 min prior to incubation with hrANXA1. The pharmacological blockage of FPR2 clearly reversed the ANXA1-mediated effect on Aβ_1–42_ phagocytosis (Fig. 5c), suggesting that ANXA1 affects microglial phagocytosis of Aβ_1–42_ by a mechanism dependent on the activation of FPR2 (two-way ANOVA, interaction *F*(6,41) = 23.04, *p* < 0.0001; effect of cell line *F*(2,41) = 13.65, *p* < 0.0001; effect of treatment *F*(3,41) = 95.70, *p* < 0.0001.

Additionally, and in order to determine if the effect of ANXA1 on microglial phagocytosis was mediated through changes in the expression of microglial receptors involved in receptor-mediated endocytosis, we measured the expression of a variety of these receptors, including the receptor for advanced glycation end-products (RAGE), the scavenger receptor macrophage receptor with collagenous structure (MARCO) and FPR in BV2 cells. Although the expression of these receptors seemed to be modified by incubation with synthetic Aβ, the effects were not reversed or modified by ANXA1 treatment (Fig. 5d–f).

### ANXA1 reduces the Aβ-induced expression of pro-inflammatory cytokines in microglia

**Fig. 6:**
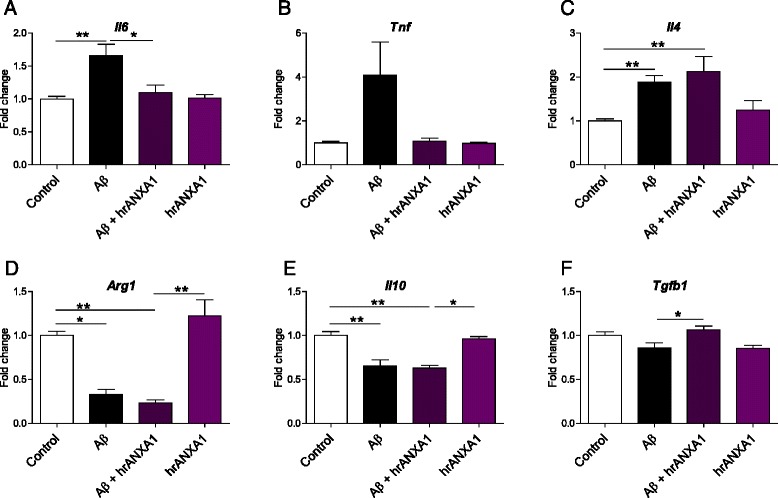
ANXA1 reduces the Aβ-induced expression of pro-inflammatory mediators in microglia. Quantification of mRNA levels of inflammatory mediators in BV2 cells treated with 3 μM Aβ_1–42_ with or without 5 μg/ml hrANXA1 for 16 h. **a** *Il6* (*n* = 6–12 samples). **b** *Tnf* (*n* = 6–17). **c** Il4 (*n* = 6–9 samples). **d** *Arg1* (*n* = 6–9 samples). **e** Il10 (*n* = 6–14 samples). **f** Tgfb1 (*n* = 6 samples). Values shown in graphs represent the mean value ± SEM and are expressed as fold change in comparison to the normalized control. Statistical analysis included one-way ANOVA with Bonferroni’s multiple-comparison post-test or Kruskal-Wallis test with Dunn’s multiple-comparison post-test, *p < 0.05, **p < 0.01

It is widely accepted that exposure of microglial cells to Aβ induces an inflammatory response, characterised by the secretion of cytokines and other pro-inflammatory molecules (see review [2]). Since ANXA1-mediated phagocytosis should work in a non-phlogistic manner, we measured the expression of pro- and anti-inflammatory mediators released during BV2 incubation with synthetic Aβ_1–42_. The levels of IL6 (*Il6*) (Fig. 6a), TNFα (*Tnf*) (Fig. 6b) and IL4 (*Il4*) (Fig. 6c) mRNAs were found increased in BV2 cells activated by synthetic Aβ_1–42_, and the expression of *Il6* and *Tnf* was clearly reduced by incubation with recombinant ANXA1. Conversely, the anti-inflammatory arginase-1 (*Arg1*), IL10 (*Il10*) and TGF-β1 (*Tgfb1*) were reduced by incubation with Aβ_1–42_ (Fig. 6d–f), and only *Tgfb1* levels were restored after exposure to hrANXA1 (Fig. 6f). Therefore, these results support the anti-inflammatory role of ANXA1 in reversing the inflammatory response induced by Aβ in microglia.

## Discussion

During the last decade, ANXA1 has gained interest as a potential pharmacological tool for the treatment of neurodegenerative disorders, including multiple sclerosis [14], stroke [31] and Parkinson’s disease [32]. Previously, we reported that ANXA1 expression is increased in microglia in close proximity to Aβ plaques using AD postmortem brain tissue [12]. In the present study, we demonstrate that brains of AD patients and a murine transgenic model of amyloidosis express higher levels of ANXA1, not only in microglia but also in astrocytes and neurons. The increases in ANXA1 observed in AD brains suggest that upregulation of ANXA1 could represent an adaptive response of microglia during inflammatory conditions and an attempt of the system to calm down the inflammation at early stages of the disease, since ANXA1 effectively solves the phase of inflammation. However, this was not observed in older animals with more severe AD pathology. This is in agreement with evidence reported in animal models of AD demonstrating that at early stages of the disease, glial cells may have a neuroprotective phenotype, secreting anti-inflammatory molecules [33, 34] and increasing Aβ phagocytosis. However, in later stages with chronic production of Aβ and pro-inflammatory cytokines, microglia change their neuroprotective phenotype in favour of a more pro-inflammatory activation state through the release of cytokines and chemokines. Interestingly, we found that the differential expression of ANXA1 in 5XFAD mice seemed to correlate with the levels of pro-inflammatory cytokines IL-6 and TNFα (Additional file 5: Figure S5E,F) and not with the expression of Aβ (Additional file 5: Figure S5G,H) or FPR (Additional file 5: Figure S5A-C). Therefore, our results indicate that the increases in ANXA1 appear to be more related to the inflammatory response following Aβ deposition rather than the levels of Aβ in the brain.

Since ANXA1 was expressed in neurons from AD patients, we investigated the in vitro effects of ANXA1 on Aβ generation and degradation using a neuronal cell line. We found that ANXA1 seems to be involved in the degradation of Aβ by neuronal cells by inducing the expression of neprilysin. Neprilysin (NEP), also known as CALLA (common acute lymphocytic leukaemia (ALL) antigen), enkephalinase, neutral endopeptidase 24.11, membrane metalloendopeptidase (MME) and CD10 antigen [35–37], belongs to the family of M13 zinc-metalloendopeptidases [38]. In the brain, NEP is considered a major amyloid-degrading enzyme [39] and is mainly located on neuronal cells, especially in the striatonigral pathway [40, 41]. In addition, it is involved in other important neuronal functions, cleaving substrates such as substance P, met- and leu-enkephalin, FMLP, the bombesin-like peptides, atrial natriuretic factor, endothelin and oxytocin [39]. Its regulation and reactivation have been extensively studied in the last decade. Factors that affect NEP expression often differ between neural and non-neural tissues as there are several NEP mRNAs which show cell- and tissue-specific expression [42]. In neurons, the neuropeptide somatostatin [43] and vitamin D are able to upregulate NEP activity [44]. Recently, it was shown that the APP intracellular domain (AICD) fragment derived from the γ-secretase cleavage of APP was also able to regulate the transcription of *NEP* [45]. Expression of the *NEP* gene is controlled through two distinct promoters [46] whose role differs between cell types, although both promoters show similar characteristics and activity. Interestingly, our study suggests that incubation of neuroblastoma cells with recombinant ANXA1 lead to an increase in NEP expression and this effect was reversed by FPR2 inhibitors. These results suggest that FPRL1/FPR2 activation by ANXA1 can regulate NEP expression, although this hypothesis needs to be further confirmed by experiments in FPRL1/FPR2 knockout cells. Similar to somatostatin receptors, FPR receptors are G protein-coupled receptors, which upon activation, trigger several agonist-dependent signal transduction pathways [47]. We do not rule out that ANXA1 could affect Aβ degradation by alternative mechanisms that do not involve FPRL1/FPR2 activation. ANXA1 can bind phospholipids in cellular membranes in a dynamic and reversible fashion in a Ca^2+^-dependent manner. This interaction can affect Ca^2+^ signalling and allows ANXA1 to contribute to the organization of membrane domains or signalling platforms and the formation of complex protein networks [48].

Additionally, we demonstrate that recombinant ANXA1 increases Aβ uptake in microglial BV2 cells and that its knockdown reduces Aβ phagocytosis. This result is in agreement with the data from Yona et al. (2006), who reported that lack of ANXA1 leads to reduced phagocytosis in ANXA1 knockout cells in the peripheral nervous system [49]. The presence of ANXA1 on the phagosomal membrane appears to be functionally important in macrophages and neutrophils. It seems, however, that the effects are more specific for Aβ_42_ than for any particle, since the phagocytosis of the scrambled Aβ peptide was less affected by ANXA1. It is well accepted that Aβ_1–42_ binds to FPRL1/FPR2 and is rapidly internalised into the cytoplasmic compartment of phagocytic cells [50]. Branderburg et al. (2008) have shown that the Aβ/FPRL1 complex co-localises with clathrin-coated endocytotic vesicles, and the activation of phospholipase D (PLD) seems to play an important role in the internalization of the Aβ/FPRL1 complex [51, 52]. In addition, FPRL1/FPR2 can interact with scavenger receptors in glial cells and this association has been reported to be important for Aβ_1–42_-mediated signal transduction [53]. One potential mechanism by which ANXA1 could affect Aβ endocytosis includes the interaction of ANXA1 with the actin cytoskeleton [54]. ANXA1 binds to and bundles F-actin in vitro and co-localises with F-actin in different cell lines [14, 55]. In vitro studies have shown that ANXA1 facilitates the interaction between F-actin and phagosomes on macrophages, whereas the knockdown of ANXA1 expression resulted in impaired phagocytosis [54]. Our results suggest that the interaction between ANXA1 and FPRL1/FPR2 is functionally important to the formation of the phagosomal membrane on microglia and Aβ phagocytosis.

In addition to the effect of ANXA1 on Aβ phagocytosis, we have shown that ANXA1 was able to reverse the pro-inflammatory effects of Aβ_1–42_ on microglia by regulating the genetic expression of *Il6*, *Tnf* and *Tgfb1*. These results are in agreement with a previous study from our group showing that ANXA1 was able to reverse the LPS-induced activation of microglia [12]. Moreover, macrophages from mice lacking ANXA1 showed higher levels of TNFα and IL-6 in response to LPS [56]. These results confirm that ANXA1 regulates microglial activation in response to inflammatory stimuli.

## Conclusions

Our data support a potential role of ANXAl in AD by reducing Aβ levels and decreasing neuroinflammation, suggesting a novel view that ANXAl may play a protective role in AD progression. We have shown in this study that ANXA1 affects the degradation and clearance of Aβ and propose ANXA1 as a promising therapeutic tool in AD. However, in spite of the protective role of ANXA1 in AD, more studies should be conducted in order to clarify possible regulatory mechanisms in this resolution pathway, which may affect its functionality in different stages of the disease.

## Additional files

Additional file 1: Figure S1. Efficiency of ANXA1 shRNA infection in BV2 cells. Representative Western blot image showing protein expression of ANXA1 in BV2 cells infected with control shRNA, and ANXA1 shRNA—clones 492A, 492B, 495A and 495B. Band intensities were determined using ImageJ and normalised to tubulin. (PDF 99 kb) Additional file 2: Supplementary methods. (DOC 22 kb) Additional file 3: Figure S3. ImageStream quantification of 5-FAM-labelled Aβ_1–42_ phagocytosis by BV2 cells. Histogram showing gating of BV2 cells. B. Scatterplot showing gating of focused BV2 cells. C. Histogram and representative images of negative and positive 5-FAM-labelled Aβ_1–42_ phagocytosis by BV2 cells. D. Representative histograms of 5-FAM-labelled Aβ_1–42_ phagocytosis by BV2 cells (WT, control shRNA and shRNA ANXA1) incubated for 3 h with 5-FAM-labelled Aβ_1–42_ (5 μg/ml) added to BV2-conditioned medium. (PDF 865 kb) Additional file 4: Figure S4. Expression of FPRL1/FPR2 in human and mouse samples and in N2asw, SK-N-SH and BV2 cell lines. A. Representative Western blots and quantification of FPRL1/FPR2 protein expression in the frontal cortex of neurologically healthy controls and sporadic Alzheimer’s patients and normalised to β-actin (*n* = 5 controls, 3 males, 2 females, range 81–97 years, mean age 86.8 ± 3 years, *n* = 7 AD cases, 3 males, 4 females, range 83–98 years, mean age 91.3 ± 2 years). B. Representative blots and quantification of FPRL1/FPR2 expression in the cortex of 5XFAD mice and wild-type littermates and normalised to β-actin (*n* = 6/group, males aged 12 and 26 weeks). C-E. Histograms showing mean intensity fluorescence (FL1) of FPRL1/FPR2 expression (red line) on (C) N2asw, (D) SK-N-SH and (E) BV2 cells analysed by FACS. Black lines shows FL1 of N2asw, SK-N-SH and BV2 cells incubated with goat anti-rabbit FITC-conjugated IgG. Values shown in graphs represent the mean value ± SEM and are expressed as fold change in comparison to the normalized control. (PDF 1076 kb) Additional file 5: Figure S5. Correlation of ANXA1 expression with FPR, cytokines and Aβ. A. Scatterplot showing relationship between FPRL1/FPR2 and ANXA1 protein expression assessed by Western blotting in the frontal cortex of neurologically healthy controls and sporadic Alzheimer’s patients (*n* = 5 controls, 3 males, 2 females, range 81–97 years, mean age 86.8 ± 3 years; *n* = 7 AD cases, 3 males, 4 females, range 83–98 years, mean age 91.3 ± 2 years). B. Scatterplot showing relationship between FPRL1/FPR2 and ANXA1 protein expression assessed by Western blotting in the motor cortex of 5XFAD mice and wild-type littermates and (*n* = 6/group, males aged 12 and 26 weeks). C. Scatterplot showing relationship between *FPR rs1* and *ANXA1* mRNA expression assessed in the frontal cortex of 5XFAD mice and wild-type littermates by qPCR (*n* = 19, males aged 12 and 26 weeks). D. Scatterplot showing relationship between ANXA1 protein expression assessed by Western blotting and TNFα expression measured by ELISA in in the frontal cortex of neurologically healthy controls and sporadic Alzheimer’s patients (*n* = 8 controls, 5 males, 3 females, range 40–82 years, mean age 67.1 ± 6 years; *n* = 10 AD cases, 7 males, 3 females, range 42–98 years, mean age 72.5 ± 6 years). E. Scatterplot showing relationship between *Tnf* and *ANXA1* mRNA expression assessed in the frontal cortex of 5XFAD mice and wild-type littermates by qPCR (*n* = 18, males aged 12 and 26 weeks). ****p* < 0.001, *r* ^2^ = 0.5779. F. Scatterplot showing relationship between *Il6* and *ANXA1* mRNA expression assessed in the frontal cortex of 5XFAD mice and wild-type littermates by qPCR (*n* = 19, males aged 12 weeks and 26 weeks). *****p* < 0.0001, *r* ^2^ = 0.6352. G. Scatterplot showing relationship between ANXA1 protein expression assessed by Western blotting and Aβ_1–40_ expression measured by ELISA in the frontal cortex of neurologically healthy controls and sporadic Alzheimer’s patients (*n* = 10 controls, 6 males, 4 females, range 40–97 years, mean age 71.5 ± 6 years; *n* = 12 AD cases, 8 males, 4 females, range 42–98 years, mean age 74.4 ± 5 years). H. Scatterplot showing relationship between Aβ and ANXA1 protein expression assessed by Western blotting in the cortex of 5XFAD mice (*n* = 11, males aged 12 and 26 weeks). (PDF 111 kb)

## Abbreviations

ABC: Avidin biotin complex
AD: Alzheimer’s disease
AICD: Amyloid precursor protein intracellular domain
ANOVA: Analysis of variance
ANXA1: Annexin A1
APP: Amyloid precursor protein
Aβ: Amyloid beta
BACE1: Beta-site APP cleaving enzyme 1
BBB: Blood-brain barrier
BSA: Bovine serum albumin
CALLA: Common acute lymphocytic leukaemia (ALL) antigen
CTF: Carboxy-terminal fragment
ELISA: Enzyme-linked immunosorbent assay
FACS: Fluorescence-activated cell sorting
FAD: Familial Alzheimer’s disease
FBS: Foetal bovine serum
FPR: Formyl peptide receptor
FPRL1: Formyl peptide receptor-like 1
hrANXA1: Human recombinant Annexin A1
HRP: Horseradish peroxidase
IDE: Insulin-degrading enzyme
IFN: Interferon
LPS: Lipopolysaccharide
MARCO: Macrophage receptor with collagenous structure
MME: Membrane metalloendopeptidase
NEAA: Non-essential amino acids
NEP: Neprilysin
NO: Nitric oxide
PBS: Phosphate buffered saline
PFA: Paraformaldehyde
PLD: Phospholipase D
RAGE: Receptor for advanced glycation end-products
ROS: Reactive oxygen species
RT-qPCR: Reverse-transcription quantitative PCR
sAPPα: Soluble APP alpha
SEM: Standard error of the mean
TBS: Tris-buffered saline
TBST: Tris-buffered saline with Tween
TNF: Tumour necrosis factor
