# Supplementary figures and images for: The anti-inflammatory Annexin A1 induces the clearance and degradation of the amyloid-β peptide
Source: J Neuroinflammation. 2016 Sep 2;13(1):234. doi: 10.1186/s12974-016-0692-6 (PMC5010757; doi:10.1186/s12974-016-0692-6)

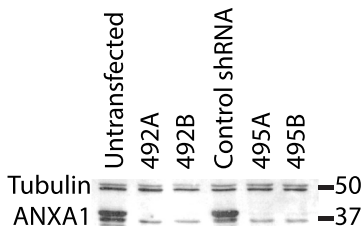

### ANXA1

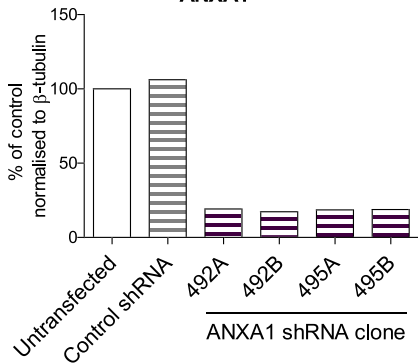

Supplement: Additional file 1: Figure S1. — Efficiency of ANXA1 shRNA infection in BV2 cells. Representative Western blot image showing protein expression of ANXA1 in BV2 cells infected with control shRNA, and ANXA1 shRNA—clones 492A, 492B, 495A and 495B. Band intensities were determined using ImageJ and normalised to tubulin. (PDF 99 kb) [file 12974_2016_692_MOESM1_ESM.pdf]

**A**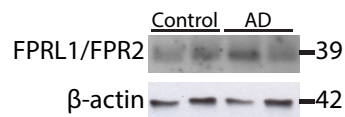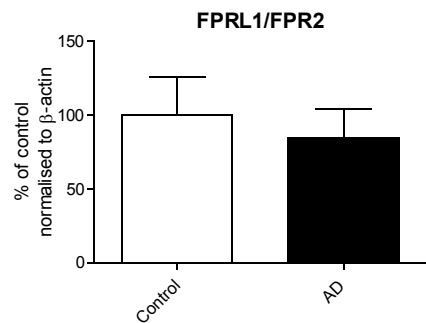**B**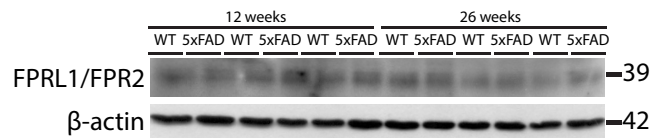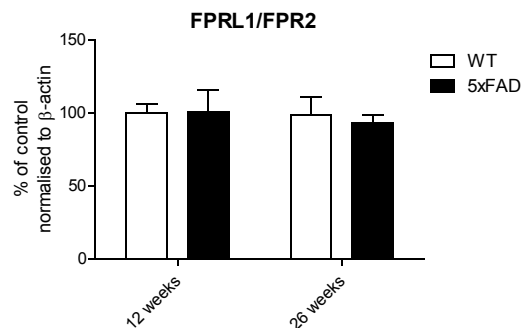**C****N2asw**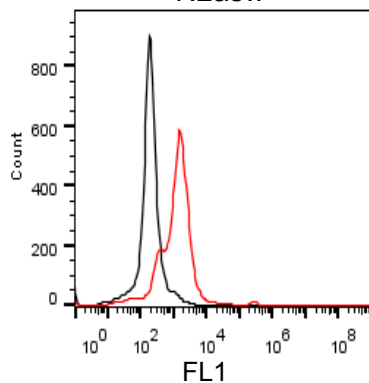**D****SK-N-SH**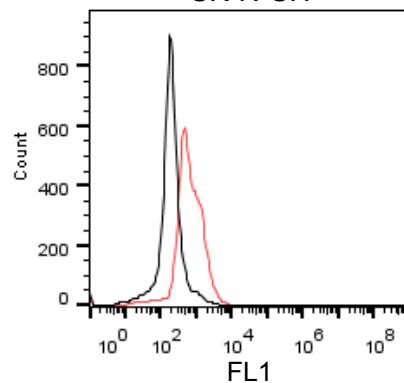**E****BV2**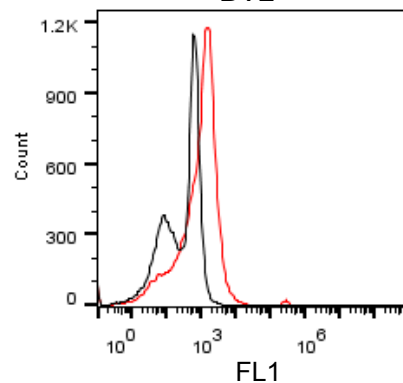

— FPRL1/FPR2  
— FITC-IgG only

Supplement: Additional file 4: Figure S4. — Expression of FPRL1/FPR2 in human and mouse samples and in N2asw, SK-N-SH and BV2 cell lines. A. Representative Western blots and quantification of FPRL1/FPR2 protein expression in the frontal cortex of neurologically healthy controls and sporadic Alzheimer’s patients and normalised to β-actin (n = 5 controls, 3 males, 2 females, range 81–97 years, mean age 86.8 ± 3 years, n = 7 AD cases, 3 males, 4 females, range 83–98 years, mean age 91.3 ± 2 years). B. Representative blots and quantification of FPRL1/FPR2 expression in the cortex of 5XFAD mice and wild-type littermates and normalised to β-actin (n = 6/group, males aged 12 and 26 weeks). C-E. Histograms showing mean intensity fluorescence (FL1) of FPRL1/FPR2 expression (red line) on (C) N2asw, (D) SK-N-SH and (E) BV2 cells analysed by FACS. Black lines shows FL1 of N2asw, SK-N-SH and BV2 cells incubated with goat anti-rabbit FITC-conjugated IgG. Values shown in graphs represent the mean value ± SEM and are expressed as fold change in comparison to the normalized control. (PDF 1076 kb) [file 12974_2016_692_MOESM4_ESM.pdf]

**A**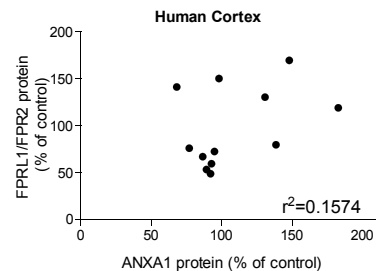**B**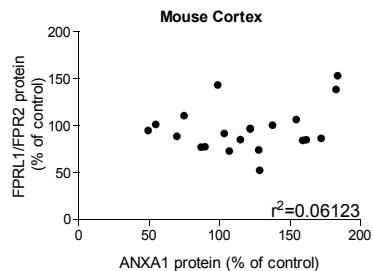**C**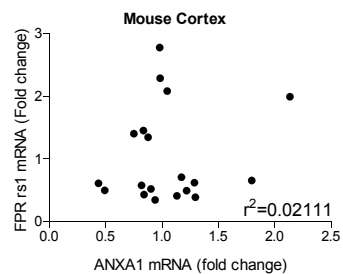**D**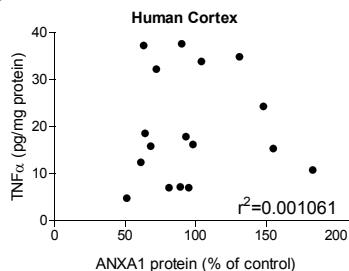**E**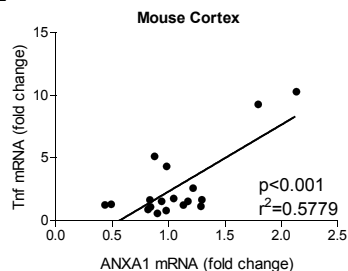**F**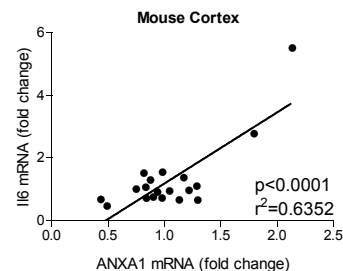**G**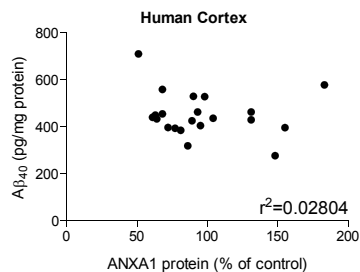**H**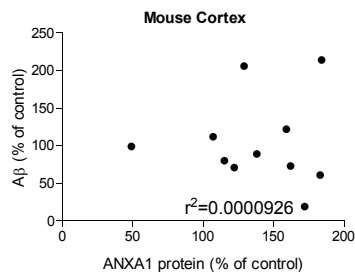

Supplement: Additional file 5: Figure S5. — Correlation of ANXA1 expression with FPR, cytokines and Aβ. A. Scatterplot showing relationship between FPRL1/FPR2 and ANXA1 protein expression assessed by Western blotting in the frontal cortex of neurologically healthy controls and sporadic Alzheimer’s patients (n = 5 controls, 3 males, 2 females, range 81–97 years, mean age 86.8 ± 3 years; n = 7 AD cases, 3 males, 4 females, range 83–98 years, mean age 91.3 ± 2 years). B. Scatterplot showing relationship between FPRL1/FPR2 and ANXA1 protein expression assessed by Western blotting in the motor cortex of 5XFAD mice and wild-type littermates and (n = 6/group, males aged 12 and 26 weeks). C. Scatterplot showing relationship between FPR rs1 and ANXA1 mRNA expression assessed in the frontal cortex of 5XFAD mice and wild-type littermates by qPCR (n = 19, males aged 12 and 26 weeks). D. Scatterplot showing relationship between ANXA1 protein expression assessed by Western blotting and TNFα expression measured by ELISA in in the frontal cortex of neurologically healthy controls and sporadic Alzheimer’s patients (n = 8 controls, 5 males, 3 females, range 40–82 years, mean age 67.1 ± 6 years; n = 10 AD cases, 7 males, 3 females, range 42–98 years, mean age 72.5 ± 6 years). E. Scatterplot showing relationship between Tnf and ANXA1 mRNA expression assessed in the frontal cortex of 5XFAD mice and wild-type littermates by qPCR (n = 18, males aged 12 and 26 weeks). ***p < 0.001, r 2 = 0.5779. F. Scatterplot showing relationship between Il6 and ANXA1 mRNA expression assessed in the frontal cortex of 5XFAD mice and wild-type littermates by qPCR (n = 19, males aged 12 weeks and 26 weeks). ****p < 0.0001, r 2 = 0.6352. G. Scatterplot showing relationship between ANXA1 protein expression assessed by Western blotting and Aβ1–40 expression measured by ELISA in the frontal cortex of neurologically healthy controls and sporadic Alzheimer’s patients (n = 10 controls, 6 males, 4 females, range 40–97 years, mean age 71.5 ± 6 years; n = 12 AD [file 12974_2016_692_MOESM5_ESM.pdf]
