# Supplementary material for: The anti-inflammatory Annexin A1 induces the clearance and degradation of the amyloid-β peptide
Source: J Neuroinflammation. 2016 Sep 2;13(1):234. doi: 10.1186/s12974-016-0692-6 (PMC5010757; doi:10.1186/s12974-016-0692-6)
Supplement: Additional file 2: — Supplementary methods. (DOC 22 kb) [file 12974_2016_692_MOESM2_ESM.doc]

Additional file 2: *Enzyme-linked immunosorbent assay* (*ELISA*). Protein expression in human cortex lysates (prepared as described above) was measured using ELISA kits for TNFα (Peprotech) and Aβ_40_ (Millipore) according to the manufacturer’s instructions. *Fluorescence-activated cell sorting*. The expression of FPRL1/FPR2 on BV2, N2asw and SK-N-SH was determined by performing a flow cytometer analysis. Briefly, cells were washed with cold PBS and fixed with 2 % PFA for 10 min. After this, cells were incubated (30 min at room temperature) with PBS containing 0.2 % BSA and rabbit anti-FPRL1/FPR2 antibody. Then, cells were washed and incubated (30 min at room temperature) with PBS containing 0.2 % BSA and goat anti-rabbit IgG FITC-conjugated (AbD Serotec) antibody. Cells were washed, centrifuged and then collected for analysis by FACS using LSR1 Fortessa equipped with four lasers: 488-nm blue laser, 561-nm yellow green laser, 641-nm red laser and 405-nm violet laser. Data were measured from the FL1 channel (mean intensity of fluorescence in log scale) with at least 10,000 events counted and analysed using FlowJo software.
