# Supplementary material for: The anti-inflammatory Annexin A1 induces the clearance and degradation of the amyloid-β peptide
Source: J Neuroinflammation. 2016 Sep 2;13(1):234. doi: 10.1186/s12974-016-0692-6 (PMC5010757; doi:10.1186/s12974-016-0692-6)

A

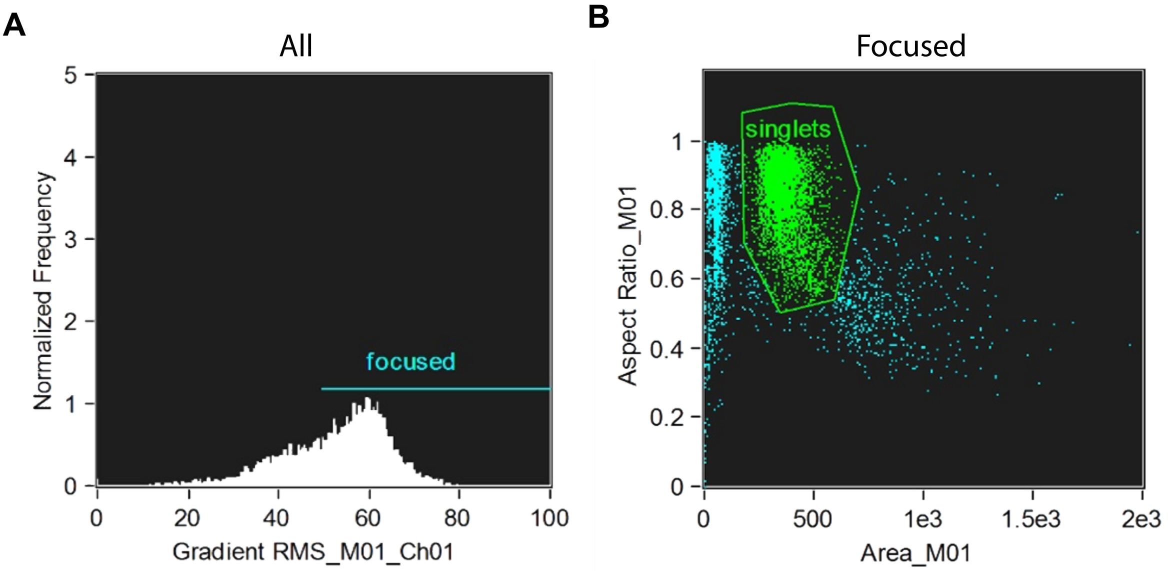

C

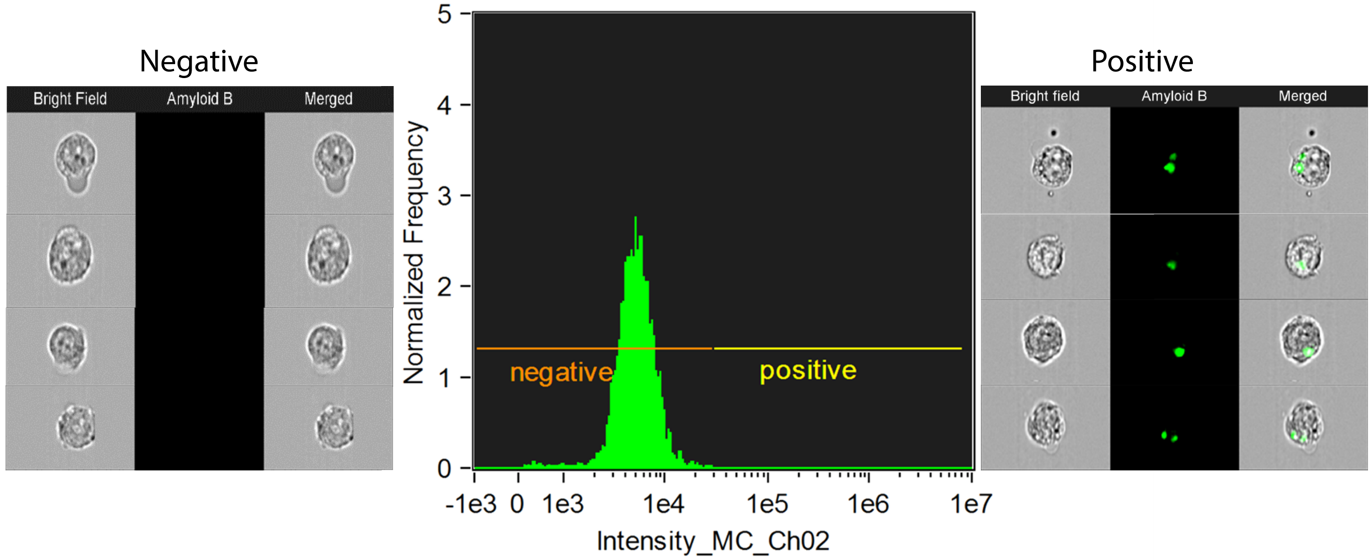

Intensity_MC_Ch02

Population	Count	%Gated
singlets & focused	5008	100
negative & singlets & focused	5006	100
positive & singlets & focused	0	0

D

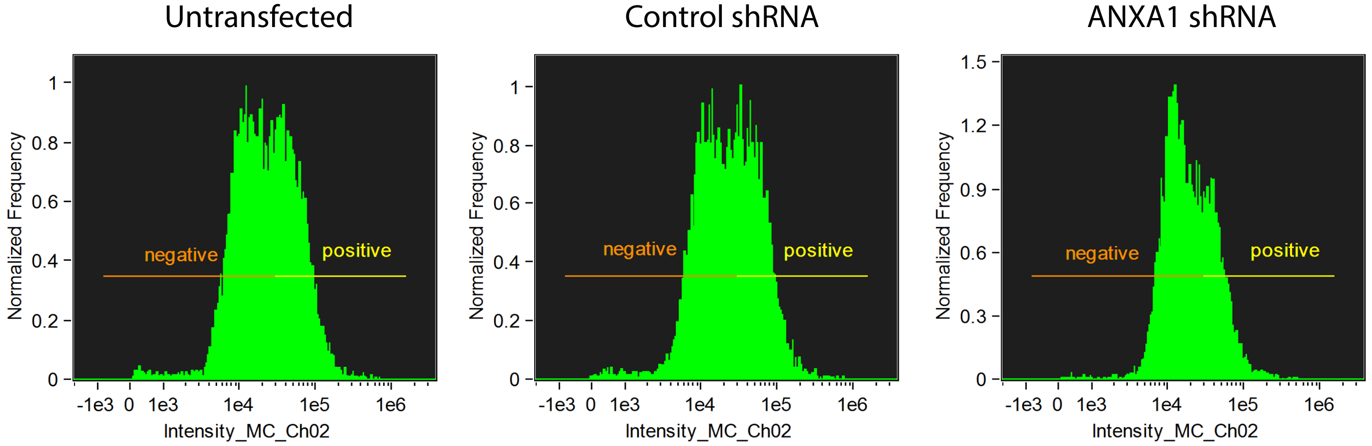

Supplement: Additional file 3: Figure S3. — ImageStream quantification of 5-FAM-labelled Aβ1–42 phagocytosis by BV2 cells. Histogram showing gating of BV2 cells. B. Scatterplot showing gating of focused BV2 cells. C. Histogram and representative images of negative and positive 5-FAM-labelled Aβ1–42 phagocytosis by BV2 cells. D. Representative histograms of 5-FAM-labelled Aβ1–42 phagocytosis by BV2 cells (WT, control shRNA and shRNA ANXA1) incubated for 3 h with 5-FAM-labelled Aβ1–42 (5 μg/ml) added to BV2-conditioned medium. (PDF 865 kb) [file 12974_2016_692_MOESM3_ESM.pdf]
